# Investigating pseudo parabolic dynamics through phase portraits, sensitivity, chaos and soliton behavior

**DOI:** 10.1038/s41598-024-64985-7

**Published:** 2024-07-02

**Authors:** Adil Jhangeer, Farheen Ibraheem, Tahira Jamal, Ariana Abdul Rahimzai, Ilyas Khan

**Affiliations:** 1 IT4Innovations, VŠB - Technical University of Ostrava, Ostrava-Poruba, Czech Republic; 2 Department of Mathematics, Namal University, 30KM Talagang Road, Mianwali, 42250 Pakistan; 3 Department of Mathematics, Forman Christian College-A Chartered University-FCCU, Lahore, Pakistan; 4 Department of Mathematics, University of the Punjab, Lahore, Pakistan; 5 Department of Mathematics, Education Faculty, Laghman University, Mehtarlam City 2701, Laghman, Afghanistan; 6 Department of Mathematics, College of Science Al-Zulfi, Majmaah University, Al-Majmaah, 11952 Saudi Arabia

**Keywords:** Oskolkov-Benjamin-Bona-Mahony-Burgers equation, Solitons, Bifurcation analysis, Revelation of chaotic dynamics, Mathematics and computing, Applied mathematics, Computational science

## Abstract

This research examines pseudoparabolic nonlinear Oskolkov-Benjamin-Bona-Mahony-Burgers (OBBMB) equation, widely applicable in fields like optical fiber, soil consolidation, thermodynamics, nonlinear networks, wave propagation, and fluid flow in rock discontinuities. Wave transformation and the generalized Kudryashov method is utilized to derive ordinary differential equations (ODE) and obtain analytical solutions, including bright, anti-kink, dark, and kink solitons. The system of ODE, has been then examined by means of bifurcation analysis at the equilibrium points taking parameter variation into account. Furthermore, in order to get insight into the influence of some external force perturbation theory has been employed. For this purpose, a variety of chaos detecting techniques, for instance poincaré diagram, time series profile, 3D phase portraits, multistability investigation, lyapounov exponents and bifurcation diagram are implemented to identify the quasi periodic and chaotic motions of the perturbed dynamical model. These techniques enabled to analyze how perturbed dynamical system behaves chaotically and departs from regular patterns. Moreover, it is observed that the underlying model is quite sensitivity, as it changing dramatically even with slight changes to the initial condition. The findings are intriguing, novel and theoretically useful in mathematical and physical models. These provide a valuable mechanism to scientists and researchers to investigate how these perturbations influence the system’s behavior and the extent to which it deviates from the unperturbed case.

## Introduction

Partial differential equations are now essential for scientists and researchers to fathom physical events due to technological developments. Advanced computational approaches have resulted in improved precision of the various physical phenomenon.

Non linear partial differential equations have proven to be useful especially for simulating non linear processes in the natural and applied sciences, such as acoustical physics, plasma physics and solid state. The aforementioned equations provide clear and comprehensive insights into the physical events under study, allowing for projections of future propagation. Furthermore, the application of non linear partial differential equations to the investigation of travelling wave features is an essential tool in many domains,like quantum physics, fluid mechanics, and several engineering specialties. As a consequence, a great deal of research has been done on studying different non linear partial differential models aiming a better understanding of the behaviour demonstrated by the physical phenomena that are being investigated. Some of the current studies have included analysis of Date-Jimbo-Kashiwara-Miwa equation^1,2^, Navier-Stokes equations^3–5^, Schr$\ddot{o}$dinger equation^6–8^, Riemann wave equation^9^, Lakshmanan-Porsezian-Daniel equation^10^, Chen-Lee-Liu dynamical equation^11,12^, and numerous other^13,14^. The exploration of soliton waves is one of the significant field in which partial differential equations of non linear form are being used more and more frequently. Localized wave pulses are recognized as solitob wave that keep propagating at the uniform speed. Researchers have been using diverse non linear models to fathom the behaviour of these waves for anticipated benefits.

Consequently, there has been a rapid growth of these waves across multiple disciplines such as non linear optics, optical fibers, ferromagnetic materials, etc. A few studies on the results of latest soliton waves can be found in^15–17^. A through understanding of soliton waves aid scientists to excel in these areas and investigate novel applications. The dynamical characteristics of the perturbed Gerdjikov-Ivanov model have been established and demonstrated by Rafiq et al.^18^. Younas et al.^19^ have studied the (2+1)-dimensional Pavlov equation by using hirota’s bilinear method to analyze the different wave structures. Bilal et al.^20^ investigated various soliton solutions of the (2+1)-dimensional soliton equation using three different analytical techniques. Bilal at al.^21^ studied Chen-Lee-Liu equation of monomode fibers by executing the logarithmic transformation, sinh-Gordon equation expansion method and the ansatz functions method along with symbolic computation. The authors achieved various types of optical soliton solutions are singular, dark, bright and their combo forms.

Pseudoparabolic equation is a nonlinear partial differential equation, that consists of a time derivative term with highest order. These equations have arisen in numerous domains Mathematics and Physics. Some eminent disciplines involve optical fiber, soil consolidation, thermodynamics, nonlinear networks, wave propagation and fluid flow within the rock discontinuities. For further information, we suggest the reader to^22,23^ and the references therein. The Oskolkov-Benjamin-Bona-Mahony-Burgers (OBBMB) equation is pseudoparabolic equation of the form

1 $$\begin{aligned} \frac{\partial p(x,t)}{\partial t}-\frac{\partial ^{3}p(x,t)}{\partial x^{2}\partial t}-k_{1}\frac{\partial ^{2}p(x,t)}{\partial x^{2}}+k_{2}\frac{\partial p(x,t)}{\partial x}+k_{3}p(x,t)\frac{\partial p(x,t)}{\partial x}=0. \end{aligned}$$

Where fluid velocity is deined by *p*(*x*, *t*) in the horizontal direction *x*, $k_{1}$ and $k_{2}$ are positive and real constant respectively and $k_{3}p(x,t)\frac{\partial p(x,t)}{\partial x}$ is a $C^{2}$- smooth nonlinear function. This pseudoparabolic one-dimensional equation being nonlinear portrays non linear surface waves that propagate along the $k_{1}p_{xx}$ and *Ox* is velocity phrase. G$\ddot{o}$z$\ddot{u}$kizi and Akçağil^24^ used tanh-coth approach and symbolic computation to obtain novel abundant solutions of OBBMB equation. Akcagil et al.^25^ have been utilized $(\frac{G^{\prime }}{G})$ expansion method to find out the analytical traveling wave solutions of OBBMB equation. Moreover, Hosseini et al.^26^ obtained analytical solutions of OBBMB equation by applying a modified Kudryashov method. Ray^27^ has been used to study the OBBMB equation by Lie symmetry analysis in order to determine symmetry reduction and compute vector fields. Aristov^28^ has examined the linear source OBBMB equation, and certain groups of solutions were described in terms of special functions. Ilhan et al.^29^ employing the $exp(-\phi (\eta ))$ expansion method in modified form generated various singular periodic and sigular soliton wave solutions that include trigonometric, hyperbolic and exponential function patterns to the OBBMB equation. Ghanbari^30^ investigated travelling wave solutions of OBBMB equation by utlizing generalized exponential rational function approach.

Bifurcation analysis applied to differential equations has been a fascinating area of research in recent times^31^. Raza et al.^32^ examined quasi periodic, periodic and super nonlinear wave phenomena in cascaded system. Jamal et al.^33^ examined the model named Novikov-Veselov and derived soliton solutions. Furthermore, they examined bifurcation analysis, chaotic and quasi periodic behaviour, multistability analysis and sensitivity analysis of the model. The perturbed and unperturbed nature of dynamical system have been explored using bifurcation analysis by many authors^34^. Jamal et al.^35^ developed soliton solutions of nerve impulse model. They implemented bifurcation and chaos theory to obtain the multistability, sensitivity analysis, chaotic and bifurcation of nerve impulse model along with external perturbation. Equilibrium points are identified using bifurcation to compute all phase portraits of dynamical system. Whereas chaos theory clarify whether the model under consideration is chaotic or not? It indicates that the solutions to physical phenomena that take place in nonlinear media either stay stable or become chaotic when we apply an external force to them.

The initial conditions exclusively govern the asymptotic behavior of autonomous dynamical systems. Four types of equilibrium behaviours include a limit circle, a tours, an equilibrium point and chaos. This research revolves around chaos theory to investigate dynamical system under discussion. There are various methods for determining chaos. In the present investigation, the most beneficial ones are emphasised. According to $\ddot{O}$zer and Akin^36^, some eminent methods are Lyapounov exponents, Phase portraits, Time series, Poincaré maps, Bifurcation diagram. and Power spectrum, Although there are a number of methods (such as the Lyapunov dimension, correlation dimension, entropy, and others) for recognising chaos, they are not frequently utilised since it can be difficult to detect chaos in real systems.

In this present study, we have examined the Oskolkov-Benjamin-Bona-Mahony-Burger equation by employing the efficient and practical approaches. Generalized Kudryashov method is used to find out the analytical solutions. The eminent competence of proposed technique is its ability to solve non linear evolution equations more naturally. Furthermore, it is observed that the exact travelling wave solution yields the solitary wave solution when the parameters are assigned particular values. The method is direct, straight forward and precise. Further, bifurcation and chaos theory are used to study the dynamics of the investigated equation. Consequently, the phase portraits of bifurcation, periodic, quasi periodic and chaotic motion are discovered. Furthermore, the multistability, lyapunov exponent and sensitivity analysis of the proposed equation are examined at several beginning conditions. All these findings are novel and have not yet been discovered. These provide a valuable mechanism to scientists and researchers to investigate how these perturbations influence the system’s behavior and the extent to which it deviates from the unperturbed case.

The paper is divided in to seven sections. Section  (2) and  (3) exhibit the algorithm of the generalized Kudryashov method and analytical solution formulation of the underlying model. Section  (4) represents physical interpretation and pictorial representation of the proposed equation. In section  (5), phase portraits at points of equilibrium of the dynamical model are displayed and examined. Several methods for detecting chaos are utilized in Section  (6) to identify the chaotic behavior of the dynamical system. In Section  (7) sensitivity profile of the considered equation is examined at different initial conditions. We present a summary of all the discoveries and conclusions drawn from the investigation in the final section.

## Algorithm of the generalized Kudryashov method

Here, we explain the generalized Kudryashov approach to find out the analytical wave solutions for nonlinear equation.

Assume that we have nonlinear equation of the form

2 $$\begin{aligned} \Psi (p(x,t),p_{x}(x,t),p_{t}(x,t),p_{xx}(x,t),...)=0, \end{aligned}$$

where *p*(*x*, *t*) is an unfamiliar function, $\Psi$ is a polynomial in *p* and its many partial derivatives, including the nonlinear terms and highest order derivative. Following are the initial phases of the generalized Kudryashov approach^37^.

*Step 1*: Utilizing the transformation $p(x,t)=\phi (\xi )$ and $\xi =\alpha x-\eta t$, the partial differential equation (non linear) takes the form of an ordinary differential equation as given below:

3 $$\begin{aligned} \Psi (\phi (\xi ),\phi ^{'}(\xi ),\phi ^{''}(\xi ),...)=0, \end{aligned}$$

*Step 2*: Assume the following form of the solution to Eq. (3)

4 $$\begin{aligned} \phi (\xi )=\frac{\sum ^{N}_{i=0}a_{i}T^{i}(\xi )}{\sum ^{M}_{j=0}b_{j}T^{j}(\xi )}, \end{aligned}$$

where $a_{i}(i=0,1,2,...,N)$ and $b_{i}(j=0,1,2,...,M)$ are constants to be examined subsequently such that $a_{N}\ne 0$ and $b_{M}\ne 0$, and $T=T(\xi )$ is the solution of ordinary differential equation

5 $$\begin{aligned} \frac{dT(\xi )}{d(\xi )}=T^{2}(\xi )-T(\xi ). \end{aligned}$$

The solution to Eq. (5) are outlined below:

6 $$\begin{aligned} T(\xi )=\frac{1}{1+Be^\xi }. \end{aligned}$$

*Step 3*: The homogeneous balance approach between the highest order derivatives and the nonlinear elements in Eq. (3) can be used to calculate the positive integers *N* and *M* in Eq.  (4).

*Step 4*: Equations (4) and  (5) are substituted into Eq. (3) to produce a polynomial in $T^{i-j}$, $(i,j=0,1,2,...)$. A set of polynomial equation is obtained by equating all terms of the same power to zero. This may be solved by software packets like Maple or Mathematica to obtain the undetermined parameters $a_{i}(i=0,1,2,...,N)$ and $b_{j}(j=0,1,2,...,M)$. As a result, we are able to solve Eq. (3) precisely.

## Computation of soliton solutions for the OBBMB equation

The generalized Kudryashov approach will be used in this subsection to identify the precise traveling wave solutions to the OBBMB Eq. (1).

7 $$\begin{aligned} p(x,t)=\phi (\xi ), ~~~\xi =\alpha x-\eta t. \end{aligned}$$

The following ordinary differential equation is generated by converting Eq. (1) employing the wave transformation (7)

8 $$\begin{aligned} (k_{2}\alpha -\eta )\phi ^{''}+\alpha ^{2}\eta \phi ^{'''}-k_{1}\alpha ^{2}\phi ^{''}+\alpha \phi \phi ^{'}=0. \end{aligned}$$

Integrating Eq. (8) with respect to $\xi$ at once yields

9 $$\begin{aligned} (k_{2}\alpha -\eta )\phi ^{'}+\alpha ^{2}\eta \phi ^{''}-k_{1}\alpha ^{2}\phi ^{'}+\frac{k_{3}}{2}\alpha \phi ^{2}=0. \end{aligned}$$

Now, taking into consideration the homogeneous balance principle among the nonlinear term $\phi ^{2}$ and the highest order linear derivative $\phi ^{''}$ in Eq. (9), we acquire $N=M+2$.

If we pick $M=1$ then $N=3$. Consequently, the solution may be expressed as

10 $$\begin{aligned} \phi (\xi )=\frac{a_{0}+a_{1}T^{1}(\xi )+a_{2}T^{2}(\xi )+a_{3}T^{3}(\xi )}{b_{0}+b_{1}T^{1}(\xi )}, \end{aligned}$$

where $T=T(\xi )$ satisfies Eq. (5) and $a_{0},a_{1},a_{2},a_{3},b_{1},b_{2}$ are parameters that are be determined. Inputting Eq. (10) into Eq. (9) along with Eq. (5), a polynomial in $T(\xi )$ is obtained. Afterwards, collecting all coefficient of $T^{j}$ with same power of *j* and setting them all to zero, we obtain a system of set of algebraic equations. By employing Maple to solve the set of algebraic equations, we procure different sets of constant numbers and use those values to find the appropriate solutions.

*Case 1*: $$a_{0}=a_{0}, a_{1}=0, a_{2}=-\frac{-12b_{0}k_{2}\alpha ^{2}}{k_{3}(6\alpha ^{2}+1)}, a_{3}=\frac{-12b_{1}k_{2}\alpha ^{2}}{k_{3}(6\alpha ^{2}+1)}, \eta =\frac{\alpha k_{2}}{6\alpha ^{2}+1}, k_{1}=-\frac{5\alpha k_{2}}{6\alpha ^{2}+1}.$$

Plugging these values in Eq. (10) along with Eqs. (7) and (6), we obtain analytical solutions of Eq. (9) as

11 $$\begin{aligned} \phi (\xi )=\frac{-12\alpha ^{2}k_{2}}{K_{3}(6\alpha ^{2}+1)(1+Be^{ax-\frac{\alpha k_{2}}{6\alpha ^{2}+1}t})^{2}}. \end{aligned}$$

*Case 2*: $$a_{0}=a_{0}, a_{1}=\frac{-24\alpha ^{2}b_{0}k_{2}}{k_{3}(6\alpha ^{2}-1)}, a_{2}=\frac{12(b_{0}-2b_{1})k_{2}\alpha ^{2}}{k_{3}(6\alpha ^{2}-1)}, a_{3}=\frac{12b_{1}k_{2}\alpha ^{2}}{k_{3}(6\alpha ^{2}-1)}, \eta =-\frac{\alpha k_{2}}{6\alpha ^{2}-1}, k_{1}=-\frac{5\alpha k_{2}}{6\alpha ^{2}-1}.$$

Plugging these values in Eq. (10) along with Eqs. (7) and (6), we obtain analytical solutions of Eq. (9) as

12 $$\begin{aligned} \phi (\xi )=-\frac{12\alpha ^{2}k_{2}(2Be^{ax+\frac{\alpha k_{2}}{6\alpha ^{2}-1}t}+1)}{K_{3}(6\alpha ^{2}-1)(1+Be^{ax+\frac{\alpha k_{2}}{6\alpha ^{2}-1}t})^{2}}. \end{aligned}$$

*Case 3*: $$a_{0}=0, a_{1}=\frac{-12b_{0}k_{2}\alpha ^{2}}{k_{3}(\alpha ^{2}-1)}, a_{2}=\frac{12(b_{0}-b_{1})k_{2}\alpha ^{2}}{k_{3}(\alpha ^{2}-1)}, a_{3}=\frac{12b_{1}k_{2}\alpha ^{2}}{k_{3}(\alpha ^{2}-1)}, \eta =-\frac{\alpha k_{2}}{\alpha ^{2}-1}, k_{1}=0$$.

Inputting these values in Eq. (10) along with Eqs. (7) and (6), we obtain analytical solutions of Eq. (9) as

13 $$\begin{aligned} \phi (\xi )=-\frac{12Be^{ax+\frac{\alpha k_{2}}{\alpha ^{2}-1}t}\alpha ^{2}k_{2}}{K_{3}(\alpha ^{2}-1)(1+Be^{ax+\frac{\alpha k_{2}}{\alpha ^{2}-1}t})^{2}}. \end{aligned}$$

*Case 4*: $$a_{0}=-\frac{12b_{0}k_{2}\alpha ^{2}}{k_{3}(6\alpha ^{2}-1)}, a_{1}=-\frac{12b_{1}k_{2}\alpha ^{2}}{k_{3}(6\alpha ^{2}-1)}, a_{2}=\frac{12b_{0}k_{2}\alpha ^{2}}{k_{3}(6\alpha ^{2}-1)}, a_{3}=\frac{12b_{1}k_{2}\alpha ^{2}}{k_{3}(6\alpha ^{2}-1)}, \eta =-\frac{\alpha k_{2}}{6\alpha ^{2}-1}, k_{1}=\frac{5\alpha k_{2}}{6\alpha ^{2}-1}.$$

Substituting these values in Eq. (10) along with Eqs. (7) and (6), we obtain analytical solutions of Eq. (9) as

14 $$\begin{aligned} \phi (\xi )=-\frac{12B\alpha ^{2}k_{2}(e^{2(ax+\frac{\alpha k_{2}}{6\alpha ^{2}-1}t)})}{K_{3}(6\alpha ^{2}-1)(1+Be^{ax+\frac{\alpha k_{2}}{6\alpha ^{2}-1}t})^{2}}. \end{aligned}$$

*Case 5*: $$a_{0}=-\frac{2b_{0}k_{2}\alpha ^{2}}{k_{3}(\alpha ^{2}+1)}, a_{1}=\frac{2(6b_{0}-b_{1})k_{2}\alpha ^{2}}{k_{3}(\alpha ^{2}+1)}, a_{2}=-\frac{12(b_{0}-b_{1})k_{2}\alpha ^{2}}{k_{3}(\alpha ^{2}+1)}, a_{3}=-\frac{12b_{1}k_{2}\alpha ^{2}}{k_{3}(\alpha ^{2}+1)}, \eta =\frac{\alpha k_{2}}{\alpha ^{2}+1}, k_{1}=0$$.

Plugging these values in Eq. (10) along with Eqs. (7) and (6), we obtain analytical solutions of Eq. (9) as

15 $$\begin{aligned} \phi (\xi )=-\frac{2\alpha ^{2}k_{2}(B^{2}(e^{ax-\frac{\alpha k_{2}}{\alpha ^{2}+1}t)})^{2}-4Be^{ax-\frac{\alpha k_{2}}{\alpha ^{2}+1}t}+1)}{K_{3}(\alpha ^{2}+1)(1+Be^{ax-\frac{\alpha k_{2}}{\alpha ^{2}+1}t})^{2}}. \end{aligned}$$

*Case 6*: $$a_{0}=-\frac{12b_{0}k_{2}\alpha ^{2}}{k_{3}(6\alpha ^{2}+1)}, a_{1}=\frac{12(2b_{0}-b_{1})k_{2}\alpha ^{2}}{k_{3}(6\alpha ^{2}+1)}, a_{2}=-\frac{12(b_{0}-2b_{1})k_{2}\alpha ^{2}}{k_{3}(6\alpha ^{2}+1)}, a_{3}=-\frac{12b_{1}k_{2}\alpha ^{2}}{k_{3}(6\alpha ^{2}+1)}, \eta =\frac{\alpha k_{2}}{6\alpha ^{2}+1}, k_{1}=\frac{5\alpha k_{2}}{6\alpha ^{2}+1}$$.

Inserting these values in Eq. (10) along with Eqs. (7) and (6), we obtain analytical solutions of Eq. (9) as

16 $$\begin{aligned} \phi (\xi )=-\frac{12\alpha ^{2}k_{2}B^{2}(e^{ax-\frac{\alpha k_{2}}{\alpha ^{2}+1}t)})^{2}}{K_{3}(6\alpha ^{2}+1)(1+Be^{ax-\frac{\alpha k_{2}}{\alpha ^{2}+1}t})^{2}}. \end{aligned}$$

## Physical interpretation and pictorial representation

**Figure 1:**
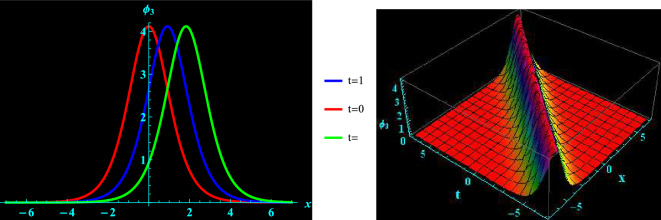
Graphical illustration of bright soliton solutions for Eq. (13) in 2D (red line corresponds to $t=0$, blue line to $t=1$, and green line to $t=2$) and 3D plots.

**Figure 2:**
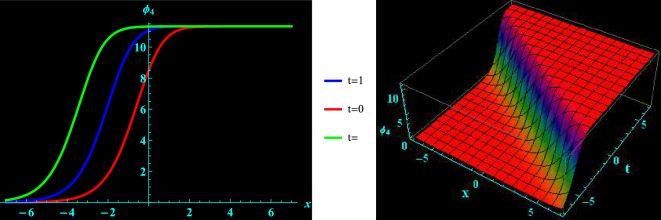
Graphical illustration of anti kink soliton solutions for Eq. (14) in 2D (red line corresponds to $t=0$, blue line to $t=1$, and green line to $t=2$) and 3D plots.

**Figure 3:**
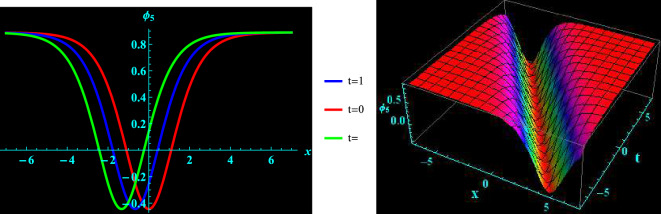
Graphical illustration of dark soliton solutions for Eq. (15) in 2D (red line corresponds to $t=0$, blue line to $t=1$, and green line to r $t=2$) and 3D plots.

**Figure 4:**
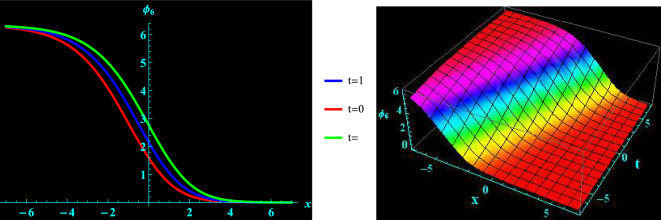
Graphical illustration of kink soliton solutions for Eq. (16) in 2D (red line correspond to $t=0$, blue line to $t=1$, and green line to $t=2$) and 3D plots.

We have demonstrated a physical justification and graphical depiction of the achieved solutions of the considered equation in this section. Let’s look at Figs. 1, 2, 3, 4, which depicts 2D and 3D representations of a few of our acquired solutions and present kink, anti kink, dark and bright soliton solutions. To do this, we select a variety of special values for the acquired parameters. For example, Fig. 1 portrays the profile of bright soliton solution of 2D and corresponding 3D shapes of Eq. (13) for $a=1.5, ~\alpha =0.7,~k_{2}=1,~k_{3}=0.7$ and $A=1$ within the interval $-10\le x\le 10$ and $-10\le t\le 10$. 2D graph for oscillation in the temporal component $t=0,1,2$ within the interval $-10\le x\le 10$ is displayed in Fig. 1. Figure 2 depicts the profile of anti kink soliton solution of 2D and corresponding 3D shape of Eq. (14) for $a=1.2, ~\alpha =-0.5,~k_{2}=-1.7,~k_{3}=0.9$ and $A=1$ within the interval $-10\le x\le 10$ and $-10\le t\le 10$. 2D graph for oscillation in the temporal component $t=0,1,2$ within the interval $-10\le x\le 10$ is shows in Fig. 2. Figure 3 represents the profile of dark soliton solution of 2D and corresponding 3D shape of Eq. (15) for $a=1.2, ~\alpha =0.2,~k_{2}=-2,~k_{3}=0.9$ and $A=1$ within the interval $-10\le x\le 10$ and $-10\le t\le 10$. 2D graph for oscillation in the temporal component $t=0,1,2$ within the interval $-10\le x\le 10$ is depicts in Fig. 3. Figure 4 illustrate the profile of kink soliton solution of 2D and corresponding 3D shape of Eq. (16) for $a=-0.7, ~\alpha =0.8,~k_{2}=-2,~k_{3}=0.5$ and $A=1$ within the interval $-10\le x\le 10$ and $-10\le t\le 10$. 2D graph for oscillation in the temporal component $t=0,1,2$ within the interval $-10\le x\le 10$ is portrays in Fig. 4.

## Bifurcation analysis

The differential equations of first order for the planar dynamical model derived from Eq. (9) are as follows:

17 $$\begin{aligned} {\left\{ \begin{array}{ll} \frac{d\phi }{d\xi }=W,\\ \frac{dW}{d\xi }=A\phi +BW-C\phi ^{2}, \end{array}\right. } \end{aligned}$$

where $A=\frac{\eta -k_{2}\alpha }{\alpha ^{2}\eta }$, $B=\frac{k_{1}}{\eta }$ and $C=\frac{k_{3}}{2\alpha \eta }$. First integral in this system is

18 $$\begin{aligned} G(\phi , W)=\frac{-A}{2}\phi ^{2}+\frac{(-B+1)}{2}W^{2}+\frac{C}{3}\phi ^{3}=k, \end{aligned}$$

where *k* takes in a number which is real. The stable points of planar dynamical model (17) on $\phi$-axis are presented by

$$T_{1}=(0,0)$$, $$T_{2}=(\frac{A}{C},0)$$.

Furthermore, the Jacobian of (17) is:

19 $$\begin{aligned} J(\phi ,W)=\begin{vmatrix} 0&1\\ A-2C\phi&B \end{vmatrix}=2C\phi -A. \end{aligned}$$

### $A>0, C>0$

**Figure 5:**
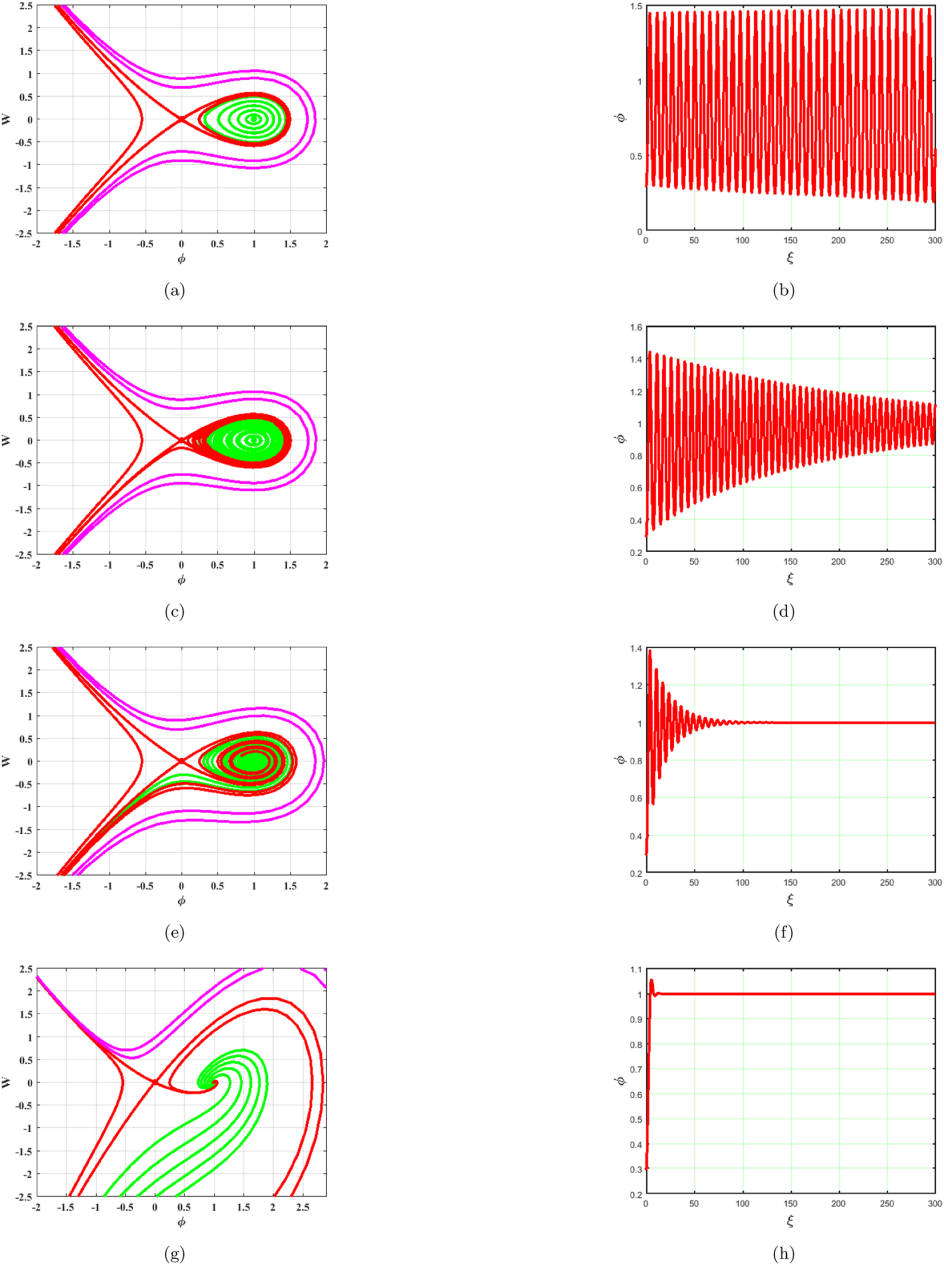
Phase portraits and respective time series plots of the planar dynamical system (17) for $B=0.0001,~0.01,~0.1,~1$, $A>0$ and $C>0$.

System (17) produces two equilibrium points, $A_{1}=(0,0)$ and $A_{2}=(1,0)$ which are shown in Fig. 5. The saddle node at $A_{1}$ and the center point at $A_{2}$ are seen in Fig. 5. Phase portraits and time series graphs are demonstrated in Fig. 5a–h respectively. As seen in Fig. 5, the term *BW* has an impact on the system (17). As $B\rightarrow 0$ system becomes stable as depicted in Fig. 5a. Different phase pictures and accompanying time series plots of the system (17) are displayed in Fig. 5 at $B=0.0001,~0.01,~0.1,~1$.

### $A<0,C<0$

**Figure 6:**
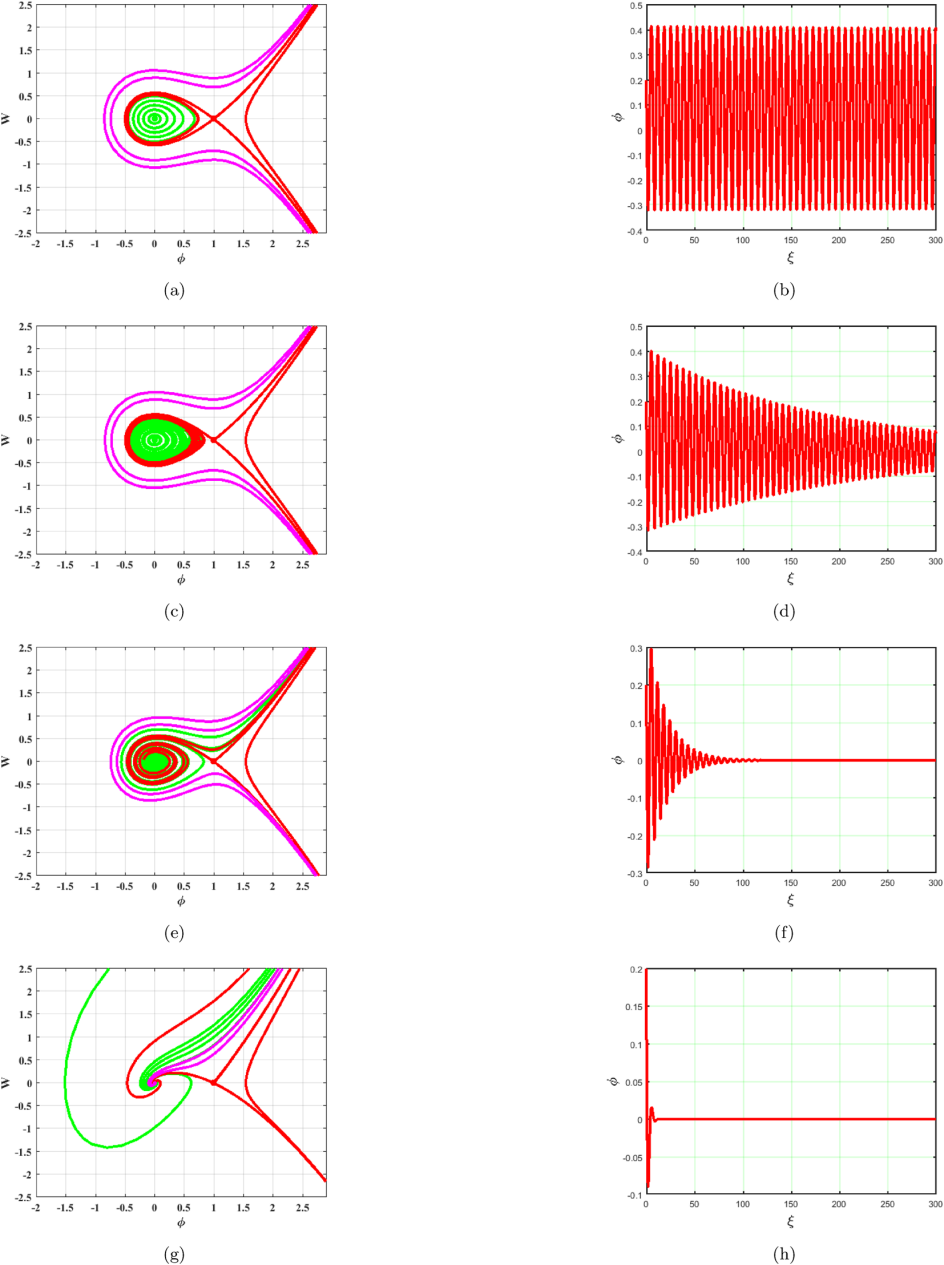
Phase portraits and respective time series plots of the planar dynamical system (17) for $B=0.0001,~0.01,~0.1,~1$, $A<0$ and $C<0$.

System (17) generates two equilibrium points, $A_{1}=(0,0)$ and $A_{2}=(1,0)$ which are illustrated in Fig. 6. The saddle node at $A_{2}$ and center point at $A_{1}$ can be observed in Fig. 6. Phase portraits and time series graphs are demonstrated in Fig. 6a–h respectively. As shown in Fig. 6, the term *BW* has an impact on the system (17). As $B\rightarrow 0$ system becomes stable as represented in Fig. 6a. Different phase pictures and accompanying time series plots of the system (17) are depicted in Fig. 6 at $B=0.0001,~0.01,~0.1,~1$.

### $A<0,C>0$

**Figure 7:**
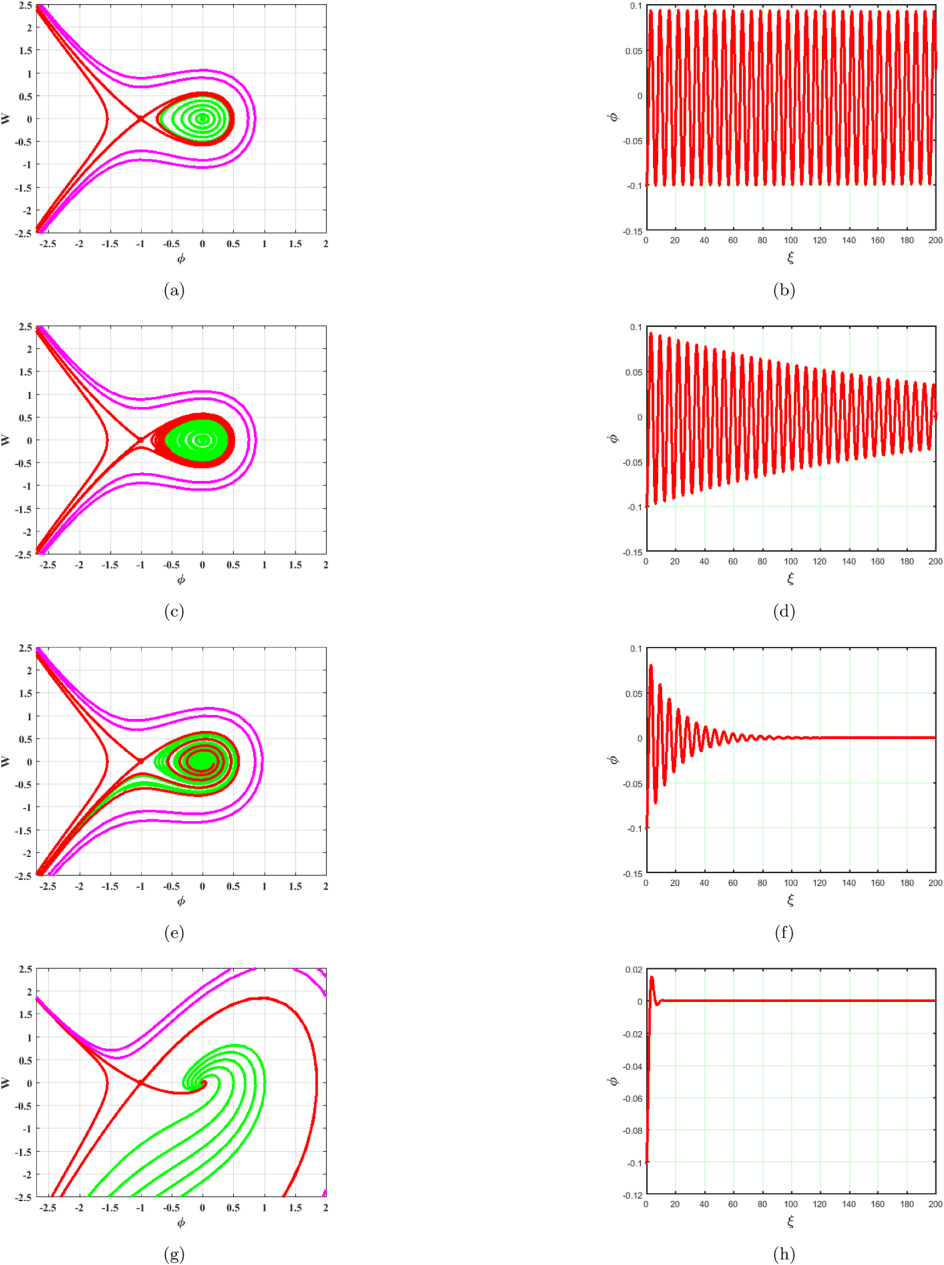
Phase portraits and respective time series plots of the planar dynamical system (17) for $B=0.0001,~0.01,~0.1,~1$, $A<0$ and $C>0$.

System (17) provides two equilibrium points, $A_{1}=(0,0)$ and $A_{2}=(-1,0)$ which are depicted in Fig. 7. The saddle node at $A_{2}$ and center point at $A_{1}$ can be viewed in Fig. 7. Phase portraits and time series graphs are displayed in Fig. 7a–h respectively. As observed in Fig. 7, the term *BW* has an impact on the system (17). As $B\rightarrow 0$ system becomes stable as represented in Fig. 7a. Different phase pictures and accompanying time series plots of the system (17) are exhibited in Fig. 7 at $B=0.0001,~0.01,~0.1,~1$.

### $A>0,\,C<0$

**Figure 8:**
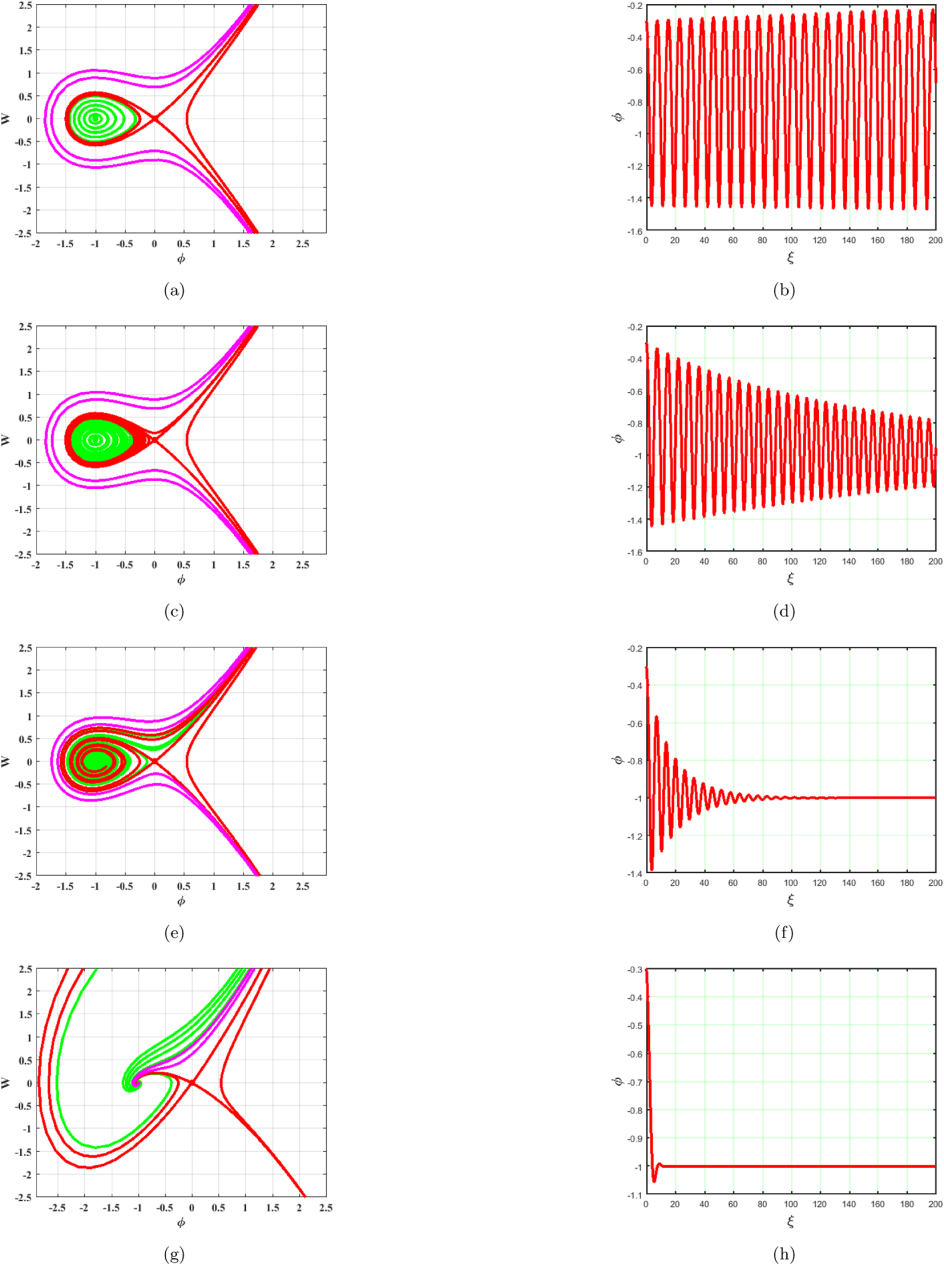
Phase portraits and respective time series plots of the planar dynamical system (17) for $B=0.0001,~0.01,~0.1,~1$, $A>0$ and $C<0$.

System (17) gives two equilibrium points, $A_{1}=(0,0)$ and $A_{2}=(-1,0)$ which can be seen in Fig. 8. The saddle node at $A_{1}$ and center point at $A_{2}$ can be noticed in Fig. 8. Phase portraits and time series graphs are given in Fig. 8a–h respectively. As illustrated in Fig. 8, the term *BW* has an impact on the system (17). As $B\rightarrow 0$ system becomes stable as displayed in Fig. 8a. Different phase pictures and accompanying time series plots of the system (17) are presented in Fig. 8 at $B=0.0001,~0.01,~0.1,~1$.

## Exploring chaotic and quasi-periodic dynamics in a perturbed dynamical system

The current section examines the investigation of the model (9), which describes chaotic and quasi-periodic behavior. In order to enhance the appeal of the planar dynamical system (17), a perturbation term called $\theta _{0}\cos (\eta \xi )$ has been introduced. Therefore, system (17) along with the perturbation term, is given as follows:

20 $$\begin{aligned} {\left\{ \begin{array}{ll} \frac{d\phi }{d\xi }=W,\\ \frac{dW}{d\xi }=A\phi +BW-C\phi ^{2}+\theta _{0}\cos (K),\\ \frac{dK}{d\xi }=\eta , \end{array}\right. } \end{aligned}$$

**Figure 9:**
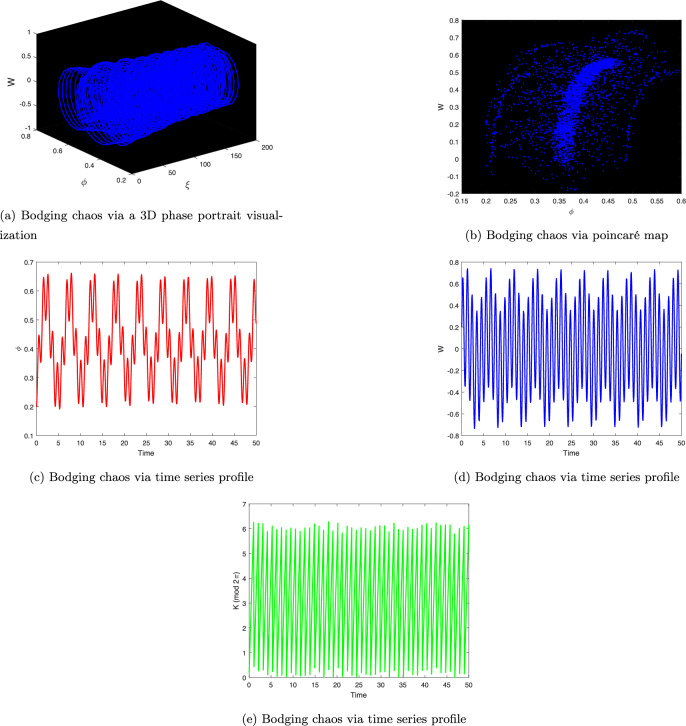
Bodging chaotic nature for model (20) via various chaos detecting mechanism with $A=1.6, B=0.0001, C=3.5, \theta _{0}=3.1, \eta =5.9$ and initial condition (0.2,0.2,0.2).

It is an independent system together with $K=\eta \xi$. The system described above utilizes the terms $\theta _{0}$ and $\eta$ to provide a disturbance term that represents the frequency and magnitude of the force that was applied, correspondingly. Whenever a system is impacted by outside forces, its behavior may change and appear random. In Fig. 9, 3D phase portrait analysis, poincaré map, and time series analysis are used to examine the chaotic behavior of the system (20). The system (20), which over time exhibits unpredictable behavior and deviates from predictable patterns, suggests chaotic dynamics.

**Figure 10:**
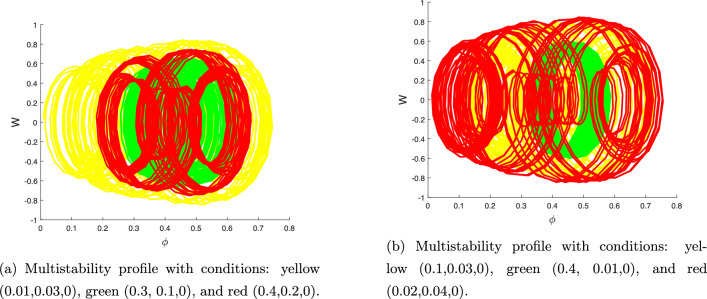
Bodging chaotic nature for model (20) via multistability profile with $A=1.6, B=0.0001, C=3.5, \theta _{0}=3.1, \eta =5.9$.

The perturbed model (20) has been investigated for multistability under various initial circumstances in Fig. 10. Observations indicate that system (20) is particularly susceptible to chaotic beginning conditions. Understanding this multistability property, which is a crucial component of complex dynamical systems, can aid in explaining and forecasting the behaviour of these systems in a variety of situations.

**Figure 11:**
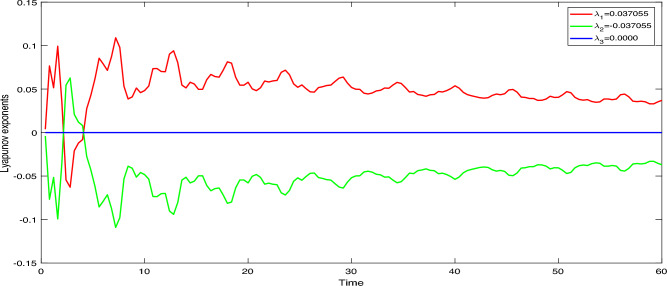
Classification of chaos in system (20) using Lyapunov exponents at $A=1.6, B=0.0001, C=3.5, \theta _{0}=3.1, \eta =5.9$ and initial condition (0.02,0.02,0.02).

On the basis of Gram-Schmidt method of orthogonalization, we applied the Wolf algorithm for the computation of Lyapunov exponents for the underlying system. Lyapunov exponents are metrics used in dynamical systems to quantify the rate at which infinitesimally close trajectories diverge or converge. They measure how small variations in the initial conditions of a system evolve over time. In essence, a Lyapunov exponent indicates the exponential rate at which nearby trajectories separate or come together in the phase space of a dynamical system. Therefore, Lyapunov exponents were introduced to measure the rate of separation between neighboring trajectories, allowing the exploration of a system’s sensitivity to initial conditions. The results show that a positive Lyapunov exponent indicates that the system has chaotic properties, meaning that even a small initial difference will cause trajectories to diverge exponentially. When the Lyapunov exponent is zero, the system is stable, and neighboring trajectories remain at a constant distance. If the Lyapunov exponent is negative, the particle orbits exhibit asymptotic stability, causing nearby trajectories to converge and overlap. So, a positive value of $\lambda =0.037055$ represents chaotic behavior of the system, while $\lambda =0$ indicates stable behavior. A negative value of $\lambda =-0.037055$ depicts asymptotic stability. To gain intricacies of the perturbed system (20), behaviour of these exponents over time have been plotted. the chaotic nature of the perturbed dynamical model (20) at $A=1.6, B=0.0001, C=3.5, \theta _{0}=3.1, \eta =5.9$, and the initial condition (0.2, 0.2, 0.2), the acquired Lyapunov exponents against time are plotted in Fig. 11.

**Figure 12:**
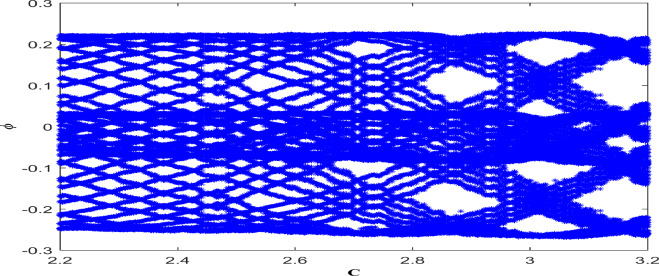
Classification of chaos in system (20) using bifurcation diagram between $\phi$ and *C* under the physical parameters $A=-1.6, B=0.0001, \theta _{0}=3.1, \eta =5.9$ under initial constraints (0.03,0.03,0.03).

The behaviour of dynamical model under the influence of parameter variation is investigated through bifurcation diagram. Specifically, critical values of parameter for scenarios such as onset of chaos, transition from stable to unstable dynamics are identified.This examination also enlightens system’s potential behaviour like limit cycle, chaos or fixed points. With physical variables $A=-1.6, B=0.0001,\theta =3.1, \eta =5.9$, and a starting condition of (0.03,0.03,0.03), the perturbed system (20) *C* versus $\phi$ has been studied via bifurcation diagram as depicted in Fig. 12 . According to the use of tools for identifying chaos, multistability analysis, time series investigation, 3D phase portrait visualisation, poincaré, the Lyapunov exponent and bifurcation diagram, the dynamical system (20) under investigation exhibits irregular, chaotic, and uncertain behaviour. Thus, this finding forms the basis of our conclusion.

## Sensitivity profile of the underlying dynamical model

**Figure 13:**
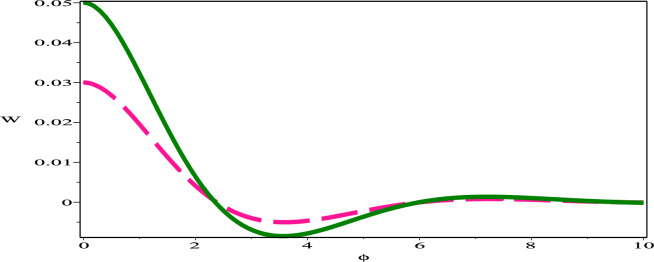
Sensitivity profile of dynamical model (17) with ($\phi$, W)=(0.05,0) in green (solid) hue and ($\phi$,W)=(0.03,0) in deep-pink (dash) hue.

**Figure 14:**
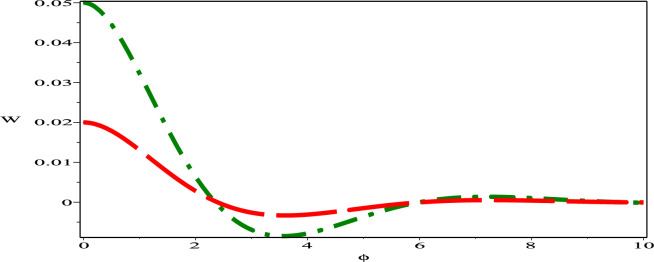
Sensitivity profile of dynamical model (17) with ($\phi$, W)=(0.05,0) in green (dash-dot) hue and ($\phi$,W)=(0.02,0) in red (long-dash) hue.

**Figure 15:**
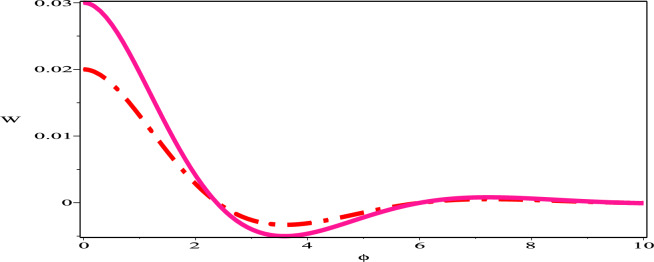
Sensitivity profile of dynamical model (17) with ($\phi$, W)=(0.03,0) in deep-pink (solid) hue and ($\phi$,W)=(0.02,0) in red (dash-dot) hue.

**Figure 16:**
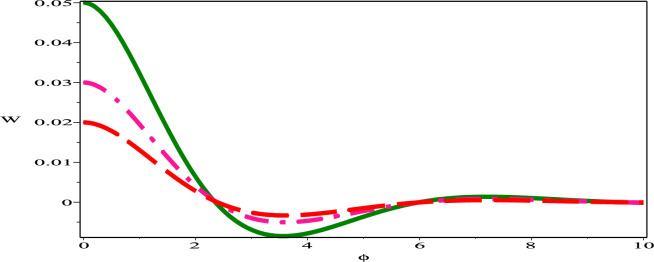
Sensitivity profile of dynamical model (17) with ($\phi$, W)=(0.05,0) in green (solid) hue, ($\phi$,W)=(0.03,0) in deep-pink (dash-dot) hue and ($\phi$,W)=(0.02,0) in red (long-dash) hue.

The sensitivity profile of the dynamical model  (17) has been built with three distinct preliminary scenarios.The two and three solution curves are investigated and compared through parameter values $A=-1, B=-1, C=1$ as displayed in figures. Fig. 13 exhibits two solutions: ($\phi$, W)=(0.05,0) in green (solid) hue and ($\phi$, W)=(0.03,0) in deep-pink (dash) hue. Fig. 14, presents two solutions: ($\phi$, W)=(0.05,0) in green (dash-dot) hue and ($\phi$, W)=(0.02,0) in red (long-dash) hue. Fig. 15 depicts two solutions: ($\phi$, W)=(0.03,0) in deep-pink (solid) hue and ($\phi$, W)=(0.02,0) in red (dash-dot) hue. Nevertheless, as Fig. 16 illustrates, a comparison was conducted at various preliminary conditions, including (0.05,0), (0.03,0) and (0.02,0). It is evident that a small variation in the starting values results in a large variation in the solution. We thus get the conclusion that the model under consideration is quite sensitive.

## Conclusion

Pseudoparabolic physical nonlinear models identified as Oskolkov-Benjamin-Bona-Mahony-Burgers (OBBMB) equation is explored. The underlying model is converted into an ordinary partial differential equation through wave transformation. Generalized Kudryashov technique is implemented to find analytical solutions that are exponential functions. Bright, anti kink, dark and kink soliton solutions are derived. Figs. 1, 2, 3, 4 exhibits 3D and 2D graphs against appropriate parametric values. The planar dynamical system (17) that resulted after Galilean transformation has been examined at the equilibrium points to conduct bifurcation analysis. It is observed that as $B\rightarrow 0$, the system (17) attains stability which is illustrated in Figs. 5, 6, 7, and 8. Further more, a periodic external perturbation term is added to obtain perturbed dynamical system (20). The chaotic nature of the model (20) is discerned through poincaré map, 3D phase portrait and time series profile as depicted in Fig. 9. This revealed the vulnerability of the system to chaotic initial conditions and Fig. 11 demonstrate plot of resulting Lyapunov exponents. With an initial condition of (0.03,0.03,0.03) and physical parameters $A=-1.6, B=0.0001, \theta _{0}=3.1, \eta =5.9$, the bifurcation diagram of the perturbed system (20) versus *C* and $\phi$ has been studied in Fig. 12. Finally, sensitivity profile has been performed with three different initial conditions. It is evident from Figs. 13, 14, 15 and 16, that the model is greatly affected by slight variation in the initial condition and exhibits significant diversions. The results that have been presented are intriguing, new, and potentially helpful in understanding how disturbances in marginally stable or unstable media evolve over time. These ramifications will make leading research much easier in the future. In conclusion, we believe that more complex nonlinear partial differential equations can be solved using this approach.

## Data Availability

Data sharing is not applicable to this article as no datasets were generated or analyzed during the current study.

## References

[1] Iqbal MA, Wang Y, Miah MM, Osman MS (2022). Study on Date-Jimbo-Kashiwara-Miwa equation with conformable derivative dependent on time parameter to find the exact dynamic wave solutions, Fractal. Fractal.

[2] Eidinejad Z, Saadati R, Li C, Inc M, Vahidi J (2023). The multiple exp-function method to obtain soliton solutions of the conformable Date-Jimbo-Kashiwara-Miwa equations. Int. Modern. Phys. B.

[3] Jamal T, Jhangeer A, Hussain MZ (2023). Propagation of velocity profile of unsteady magnetohydrodynamics flow between two orthogonal moving porous discs. Eur. Phys. J. Plus.

[4] Hess MW, Quaini A, Rozza G (2023). A data-driven surrogate modeling approach for time-dependent incompressible Navier-Stokes equations with dynamics mode decomposition and manifold interpolation. Adv. Comput. Math..

[5] Lange, T. Regularization by noise of an averaged version of the Navior-Stokes equatioms, *J. Dynam. Differ. Equ.* 1-26, (2023). 10.1007/s10884-023-10255-5 PMC11564229 39554540

[6] Skipp J, Laurie J, Nazarenko S (2023). Hamiltonian derivation of the point vortex model from the two-dimensional nonlinear Schrödinger equation. Phys. Rev. E.

[7] Wang KJ, Liu JH (2023). Diverse optical solitons to the nonlinear Schrödinger equation via two novel techniques. Eur. Phys. J. Plus.

[8] Asjad MI, Inc M, Fraidi WA, Bakar MA, Muhammad T, Rezazadeh H (2023). Optical solitonic structures with singular and non-singular kernel for nonlinear fractional model in quantum mechanics. Opt. Quant. Electron..

[9] Muhamad KA, Tanriverdi T, Muhamud AA, Baskonus HM (2023). Interaction characteristics of the Riemann wave propagation in the (2+1)-dimensional generalized breaking soliton system. Int. J. Comput. Math..

[10] Wu XH, Gao YT, Yu X, Ding CC, Li LQ (2022). Modified generalized Darboux transformation and solitons for a Lakshmanan-Porsezian-Daniel equation. Chaos. Soli. Fract..

[11] Kumar S, Niwas M (2023). New optical soliton solutions and a variety of dynamical wave profiles to the perturbed Chen-Lee-Liu equation in optical fibers. Opt. Quant. Electron..

[12] Faridi WA, Asjad MI, Jarad F (2022). Non-linear soliton solutions of the perturbed Chen-Lee-Liu model ny $$\Phi ^{6}$$-model expansion approach. Opt. Quant. Electron..

[13] Baber MZ, Seadway AR, Iqbal MS, Ahmad N, Yasin MW, Ahmad MO (2023). Comparative analysis of numerical and newly constructed soliton solutions of stochastic Fisher-type equations in a sufficiently long habitat. Int. J. Modern Phys. B..

[14] Liu JG, Osman MS (2022). Nonlinear dynamics for different nonautonomous wave structures solutions of a 3D variable-coefficient generalized shallow water wave equation. Chin. J. Phys..

[15] Aksoy A, Yenikaya S (2023). Soliton wave parameter estimation with the help of artificial neural network by using the experimental data carried out on the nonlinear transmission line. Chaos Solit. Fract..

[16] Khater MM (2023). A hybrid analytical and numerical analysis of ultra-short pulse phase shifts. Chaos Solit. Fract..

[17] Ozdemir N, Secer A, Bayram M (2023). Extraction of soliton waves from the longitudinal wave equation with local M-truncated derivatives. Opt. Quant. Electron..

[18] Rafiq MH, Jhangeer A, Raza N (2023). The analysis of solitonic, supernonlinear, periodic, quasiperiodic, bifurcation and chaotic patterns of perturbed Gerdjikov-Ivanov model with full nonlinearity. Commun. Nonlinear Sci. Numer. Simul..

[19] Younas U, Ren J, Sulaiman TA, Bilal M, Yusuf A (2022). On the lump solutions, breather waves, two-wave solutions of the (2+1)-dimensional Pavlov equation and stability analysis, Moder. Phys. Lett. B..

[20] Bilal M, Rehaman SU, Ahmad J (2022). Dispersive solitary wave solutions for the dynamical soliton model by three versatile analytical mathematical methods. Eur. Phys. J. Plus.

[21] Bilal M, Hu W, Ren J (2021). Different wave structures to the Chen-Lee-Liu equation of the monomode fibers and its modulation instability analysis. Eur. Phys. J. Plus.

[22] Korpusov MO, Sveshnikov AG (2008). Blow-up of solutions of strongly nonlinear equations of pseudoparabolic type. J. Math. Sci..

[23] Dubey SA (2010). Numerical solution for nonlocal Sobolev-type differential equations. Electron. J. Differ. Eq. Conf..

[24] Gözükizi OF, Akçağil S (2013). The tanh-coth method for some nonlinear pseudoparabolic equations with exact solutions. Adv. Differ. Eq..

[25] Akcagil S, Aydemir T, Gozukizil OF (2016). Exact travelling wave solutions of nonlinear pseudoparabolic equations by using the $$(\frac{G^{\prime }}{G})$$ expansion method. New. Trend. Math. Sci..

[26] Hosseini K, Bejarbaneh EY, Bekir A, Kaplan M (2017). New exact solutions of some nonlinear evolution equations of pseudoparabolic type. Opt. Quantum. Electron..

[27] Ray SS (2020). Lie symmetries, exact solutions and conservation laws of the Oskolkov-Benjamin-Bona-Mahnoy-Burgers equation, Modern. Phys. Lett. B..

[28] Aristov AI (2018). On exact solutions of the Oskolkov-Benjamin-Bona-Mahony-Burgers equation. Comput. Math. Math. Phys..

[29] Ilhan OA, Bulut H, Sulaiman TA, Baskonus HM (2018). Dynamics of solitary wave solutions in some nonlinear pseudoparabolic models and Dodd-Bullough-Mikhailov equation. Indian J. Phys..

[30] Ghanbari B (2021). New analytical solutions for the Oskolkov-type equations in fluid dynamics via a modified methodology. Result. Phys..

[31] Liu H, Yang H, Liu N, Yang L (2022). Bifurcation and chaos analysis of tumor growth. Int. J. Biomath..

[32] Raza, N., Jhangeer, A., Arshed, S., & Inc, M. The chaotic, supernonlinear, periodic, quasiperiodic wave solutions and solitons with cascaded system, *Waves Random Compl. Media* 1-15, (2021).

[33] Jamal T, Jhangeer A, Hussain MZ (2023). Analysis of nonlinear dynamics of Novikov-Veselov equation using solitonic solutions, bifurcation, periodic and quasi-periodic solutions, and Poincaré section. Eur. Phys. J. Plus.

[34] Saha A (2017). Bifurcation, periodic and chaotic motions of the modified equal width-Burgers (MEW-Burgers) equation with external periodic perturbation. Nonlinear Dyn..

[35] Jamal T, Jhangeer A, Hussain MZ (2014). An anatomization of pulse solitons of nerve impulse model via phase portraits, chaos and sensitivity analysis. Chin. J. Phys..

[36] Özer AB, Akin E (2005). Tools for detecting chaos. SA Fen. Bilimleri. Enstits. Dergisi..

[37] Demiray ST, Pandir Y, Bulut H (2014). The investigation of exact solutions of nonlinear time-fractional Klein-Gordon equation by using generalized Kudryashov method. AIP Conf. Proc..

